# Psychosocial burden of neglected tropical diseases in eastern Colombia: an explorative qualitative study in persons affected by leprosy, cutaneous leishmaniasis and Chagas disease

**DOI:** 10.1017/gmh.2021.18

**Published:** 2021-06-18

**Authors:** Robin van Wijk, Lena van Selm, Martha C. Barbosa, Wim H. van Brakel, Mitzi Waltz, Karl Philipp Puchner

**Affiliations:** 1 NLR, Wibautstraat 137k, 1097 DN Amsterdam, the Netherlands; 2 German Leprosy and TB Relief Association, DAHW América del Sur, Calle 128 B No. 56 C 05, Bogotá, Colombia; 3 Vrije Universiteit Amsterdam, De Boelelaan 1085, 1081 HV Amsterdam, the Netherlands; 4 German Leprosy and TB Relief Association, Raiffeisenstraße 3, 97080 Würzburg, Germany; 5 Global Health–Disaster Medicine, Medical School, National and Kapodistrian University of Athens, Mikras Asias 17, Athens 115 27, Greece

**Keywords:** Chagas disease, cutaneous leishmaniasis, leprosy, mental health, social participation, stigmatization

## Abstract

**Background:**

Leprosy, cutaneous leishmaniasis (CL) and Chagas disease (CD) are neglected tropical diseases (NTDs) with a high psychosocial burden in Norte de Santander and Arauca in Colombia. This study provides insights into affected persons' feelings, perceptions and experiences to better understand the nature of this burden.

**Methods:**

In 2018, 34 leprosy, CD and CL patients participated in four focus groups discussing the influence of the disease on mental well-being, social participation and stigma. Additionally, 13 leprosy patients participated in semi-structured interviews to further explore the health-related stigma related to this disease. Audio recordings were transcribed verbatim, and open coding was used to identify the most relevant categories and themes.

**Results:**

Persons suffering from CD reported that their mental distress was mainly caused by impairments and stress related to the progressive and incurable nature of the disease. Persons affected by CL perceived the treatment for the disease as having the most impact on their psychosocial well-being. Persons affected by leprosy reported suffering most from anticipated and experienced stigma.

**Conclusions:**

The findings indicate that these diseases are likely to impose a significant psychosocial burden on patients in the studied regions, even though these vary per condition. Consistent data collection on the psychosocial burden and the sharing of knowledge of effective interventions can contribute to the holistic approach needed to win the fight against NTDs.

## Introduction

Neglected tropical diseases (NTDs) are estimated to affect over 1 billion people. Norte de Santander and Arauca are regions of Colombia that exhibit extremely high endemicity of three NTDs: Chagas disease (CD), cutaneous leishmaniasis (CL) and leprosy (Cardona-Castro *et al*., 2005; Stanaway and Roth, 2015; OPS, 2019). Colombia achieved a prevalence of leprosy below 1/10 000 over a decade ago (Cardona-Castro, 2013). However, the slow decline in incidence and the relatively high percentage of cases with disabilities upon diagnosis are indicative of a significant remaining disease burden (Padilla *et al*., 2017; OPS, 2019). In 2016, the incidence of leishmaniasis in Colombia was 53/100000; 98% of these cases had CL (OPS and WHO, 2018). Between 2008 and 2016, 7172 cases of CD were reported in Colombia, with an annual incidence of 11/100 000 (Padilla *et al*., 2017).

NTDs cause physical impairments, which may become irreversible when affected persons lack access to health care and experience detection delays (Mackey *et al*., 2014). These impairments often result in social disadvantage and potentially prevent affected persons from participating in key aspects of life, which further increase the disease burden (Sun and Amon, 2018). The full burden of NTDs is thus not merely physical but is the result of multiple factors, including stigma, social exclusion and mental distress. These factors are associated with a lower quality of life and can contribute to the aggravation of the disease itself, sustaining a highly complex chronic morbidity (Hofstraat and van Brakel, 2016).

Both leprosy and CL can lead to permanent skin lesions, disfigurement and physical disability and are strongly associated with social stigma, decreased self-esteem, decreased social participation and mental distress (Ozaki *et al*., 2011; Van Brakel, 2014; Al-Kamel, 2017). Although CD does not lead to visible impairments or disfigurement, it is also highly stigmatizing. Because cardiac complications occur in a subfraction of patients in the chronic stage of disease, CD is widely believed to reduce one's productivity and well-being. This causes a societal perception of affected people as weak and worthless (Van Brakel, 2014). Serological testing is often demanded by employers, which translates into exclusion from the labor market, causing mental distress and lower social participation (Stanaway and Roth, 2015).

The total disability-adjusted life years associated with NTDs are high: 26 billion globally. There is considerable evidence that their impact is significantly underestimated, as disease consequences such as stigma and mental distress are not routinely taken into account (Mieras *et al*., 2016; Mitra and Mawson, 2017). Our related study conducted among patients with CD, CL and leprosy in this area suggests the existence of a substantial psychosocial burden (PSB) (Gómez *et al*., 2020, p. 5): ‘In total, 31.6% (96/304) of the study participants exhibited a moderate or high PSB. The highest percentage was 41.5% (44/106), found in the leprosy group, followed by 25% (25/100) in the CD group’. However, this quantitative study did not explore feelings, perceptions and experiences in depth. Therefore, the present study serves as a complementary qualitative approach to better understand the context of the information obtained. The objective of this study was to explore what factors contribute to the PSB of persons affected by CD, CL and leprosy in two endemic areas in Colombia. Based on the findings of Gómez *et al*. (2020), the scope of social participation restriction was further examined, complemented by an in-depth study on experienced stigma among persons affected by leprosy. This information is urgently needed to comprehend the full psychosocial dimensions and consequences of NTDs and to support advocacy for increased funding and the implementation of available disease management, disability prevention and inclusion interventions (Mieras *et al*., 2016).

## Materials and methods

### Study design

This qualitative study adopts an explorative approach to determine the dimensions of stigma, mental distress and participation restriction related to the PSB experienced by people affected by CD, CL or leprosy in two regions of Colombia.

### Study setting

The study was conducted in Norte de Santander and Arauca between May and June 2018. Life expectancy at birth in both departments is 70 years (Knoema, 2005). Sixty-four percent of the population in Arauca and 77% in Norte de Santander live in urban areas. According to Cardona-Castro (2013), high detection rates for leprosy are found in Arauca (4.73/10 000) and Norte de Santander (3.86/10 000). The prevalence of CD in both departments ranges from 0.1% to >15% at the sub-district level (Olivera *et al*., 2019). The incidence of CL in Arauca is relatively low, 0–0.31/100.000, but is much higher in Norte de Santander at 2.33–18.13/100 000 (OPS and WHO, 2018).

### Study population

Adults (⩾18 years old) with a diagnosis of leprosy, CL or CD reported to the national NTD program and living in Norte de Santander or Arauca were eligible for this study. Study participants were recruited among the study population of the related quantitative study by Gómez *et al*. (2020), participants were asked to also participate in focus group discussions (FGDs) or interviews. From this emerged a convenience sample based on willingness and availability rather than on variability in the population. The population was first classified by disease type. Subsequently, participants were admitted until a sufficient number of participants for the FGDs and interviews was reached across all three conditions.

Based on the finding of Gómez *et al*. (2020) that experienced stigma was significantly higher among persons affected by leprosy compared to CL and CD, additional interviews with persons affected by leprosy were facilitated by the Norte de Santander Institute for Health to obtain in-depth information on the impact of stigma on mental well-being and social participation. Interviews were conducted until data saturation was reached.

### Focus group discussion

FGDs were set up to detect similarities and differences among participants affected by the three NTDs, based on the domains of life found by Eyssen *et al*. (2011): work/study, social life, general participation and home (Appendix A). The FGD guides were prepared in Spanish, and each FGD was moderated by an experienced senior researcher from the Colombian research team, assisted by two other members of the research team, who helped to maintain the FGD structure and took notes. The topic guide consisted of three parts (Green and Thorogood, 2014): participants were invited to tell a personal story related to family, work and community (engagement question), after which the three most important stories agreed on by the group were further discussed (exploration question) and explored in-depth during the FGD. Finally, participants were invited to contribute anything they thought would also be important (exit question).

### Interviews

The interviews consisted of five predefined questions based on Weiss's framework for the assessment of health-related stigma (Appendix B; Weiss, 2008). The interview guides were prepared in Spanish. The following categories measured from the perspective of the affected person were included: anticipated stigma, experienced stigma and self or internalized stigma. Anticipated stigma refers to stigma regarded as unjustified but likely. Internalized stigma refers to the process where patients accept the perceived exclusionary views of society and stigmatizes themselves. Furthermore, follow-up questions were asked based on the experiences shared by the participants and which often also included aspects of mental well-being and social participation. The interviews were conducted in Spanish by a Colombian research assistant, guided and assisted by a non-native Spanish-speaking researcher.

### Ethical considerations

This study was approved by the Ethics Committee of Francisco de Paula Santander University in the city of Cúcuta by means of Act No. 2 of 21 November 2017. All participants signed an informed consent form.

### Analysis

Audio-recorded data were transcribed verbatim by two research assistants. The FGD moderator and the interviewer reviewed the transcripts. A unique identifying number was assigned to each participant to ensure anonymity. Then, the initial coding started. The codes were short tags of information derived from the transcripts using Atlas.ti, version 8. An open coding method was used; in this way, many different codes were formulated. Using these codes, subdivisions were made for different topics and themes. Themes regarding mental well-being, participation and stigma were identified and related to Weiss's (2008) study on stigma and the theoretical study of Eyssen *et al*. (2011) on life domains. Findings in the FGDs and interviews that validated or contradicted one another were matched to create data triangulation.. Findings were discussed with the native Spanish-speaking researchers and agreement was reached on the included results and the interpretation and translation.

## Results

### Study population

Four FGDs were held between May and June 2018, with a combined group of 6–11 persons affected by one of the three diseases. In every group, representatives from two diseases were present (see Table 1 for demographic details). A limited number of persons affected by CD participated in the FGDs in Norte de Santander; therefore, additional participants for a fourth FGD were recruited in Arauca.

**Table 1.** Demographic information research population FGD participants

	Group 1	Group 2	Group 3	Group 4
Participants	11	10	6	7
Diseases	Leprosy: 8 CD: 3	Leprosy: 8 CD: 2	CL: 5 CD: 1	CD: 6 Leprosy: 1
Sex	Male: 3 Female: 8	Male: 6 Female: 4	Male: 5 Female: 1	Male: 7
Mean age (years)	52 s.d.: 9.6	60 s.d.: 9.3	49 s.d.: 8.2	53 s.d.: 5.1
Urban/rural	Urban: 10 Rural: 1	Urban: 8 Rural: 2	Urban: 2 Rural: 4	Urban: 6 Rural: 1

s.d., Standard deviation.

In total, 13 semi-structured interviews were conducted. All respondents were persons affected by leprosy, between 28 and 69 years old, with a mean age of 56. Seven were male, and six were female. All interviewees lived in urban areas. Since data saturation had been reached in Norte de Santander and a sufficient number of persons affected by leprosy had participated, no additional interviews were conducted in Arauca. The most important findings are presented in the themes below, with references to quotes identified according to the group/interview the topic was discussed in (Group 1 = G1, etc.; Interview 1 = I1, etc.).

### Mental well-being

#### Support

Although not all FGD and interview participants received the same level of support from their families, they agreed that support is important to be able to cope emotionally with diagnosis, impairment and treatment for their disease. Participants appreciated it when their family was concerned about them: *‘My husband watched over me; he came to the clinic every day…’* [G3 – F, 54, CL].

Some of the FGD participants mentioned that they had become closer to their families due to their diseases and that they had learned more about their families and the strength of their bonds. In FGD 4, participants explicitly mentioned that their families were worried that they could die from the heart condition caused by CD.

FGD and interview participants said practical support was also important. Family members who reminded them to go to appointments and take medication were said to be very helpful, and these practical actions made them feel supported: ‘*My children were the ones who encouraged me most: no, Dad, you have to fight’* [I3 – Male, 59, leprosy].

Some people affected by CL stated in the FGDs that the treatment is more difficult to cope with, both physically and emotionally, than the disease. People affected by leprosy depended more on their families: ‘*In my house lives a lot of family, and thank God, there hasn't been rejection … we all helped each other, and there hasn't been any discrimination, nothing’* [G1 – F, 48, leprosy].

#### Anxiety

In FGD 4, stories were shared about how CD in the community can be worrying:

*‘I told a friend that I had Chagas disease, and he was worried; he told me:’ ‘Brother, careful with this Chagas – you need to be attentive because I used to have a friend who got Chagas, and within two months, he died; he was 28 years old’* [G4 – M, 46, CD].

Some FGD participants suffering from CD-linked heart problems mentioned that they live in constant fear of death. Every time they feel their heart is having difficulties, they are afraid something bad will happen. One FGD participant who was infected with CL as a child explained how he was traumatized by the intensity of the medication: ‘*The treatment is tough, the treatment is not so easy; that is what I think is psychologically most affecting’* [G3 – M, 40, CL].

During the interviews with persons affected by leprosy, the majority (*n* = 9) mentioned they experienced negative emotions when they got the diagnosis. They felt scared, bad and sad. Two of them felt life was not worth living anymore. The patients who felt scared mentioned that they did so because they had heard about people with leprosy being isolated; they thought it was untreatable and feared they would be rejected by others: ‘*I said, “Fine, now the whole world is going to reject me, my friendships are going to end. Well, everything!”’* [I13 – M, 64, Leprosy]

Patients without negative emotions as a result of the diagnosis explained this was either because they were children at the time of diagnosis or because they did not know about the disease before and the doctor was able to reassure them.

### Social participation

#### Family

In FGD 2, one man with leprosy said he was not allowed to eat with the rest of the family because his father and brother did not want their plates and food to be mixed with his. Likewise, in the interviews, a man reported he had to eat separately with his own plate and do his own dishes, and his wife would become upset with him when he touched other things. A woman shared that she had cared for her husband while he was ill, but when she was diagnosed with leprosy and started treatment, he let her down by leaving her alone and in debt.

FGD participants with CL shared a perception of inequality due to functional impairment and shared views regarding the consequences for family life. They expressed that families suffered financially due to of a loss of household income and emotionally due to the burden of taking care of a sick family member. One FGD participant affected by CL explained he had never felt rejected by his family but had difficulties with accepting his (temporary) disabilities and did not feel equal to other family members because he could not contribute to the household income or do chores anymore.

#### Work

Misconceptions and functional impairments were mentioned by FGD participants with leprosy in both the first and second groups. Their colleagues started to avoid them because they were afraid of getting infected. Two people affected by leprosy had lost their jobs because they could not use their hands and feet properly, which made them unable to perform their work. Misconceptions and employers' fear of transmission also influenced the continuation of employment:

I told the doctor what to discuss because they [the participant's employers] were going to throw me out, and that would be terrible. The doctor went to see them and told them no, that is not bad, that it is normal, there is a treatment and nothing is going to happen. And in the end, everything worked out well [G1 – F, 49, leprosy].

FGD participants affected by CL and CD also reported a major impact on participation in the workplace. Those affected by CL stated that this was due to the fatigue and the musculoskeletal pain they experienced as a result of their treatment. During this period, which varied from 20 days to 3 months, they physically struggled to perform their jobs:

Imagine when an employee would be saying: ‘No, these 20 days during treatment I can't work for you,’ that will make them fire you very fast … we are talking about the countryside, not about some company where everything is within the law; at the countryside, when you make a long face once and they don't like that: ‘Bye!’ [G3 – M, 61, CL]

In participants with CD, the physical restrictions related to work were caused by the treatment as well as chronic disease symptoms. All FGD participants with CD in this group agreed that the side effects of the treatment were detrimental to their working life. Those affected by cardiac complications had to stop working or adjust their work. This was a longer-term process and was perceived as a personal short-coming: ‘*You wish you could do things, but you can't … at times, one wants to do more but cannot, so that gives a feeling of incapability. Frustration and incapability’* [G4 – M, 56, CD].

### Stigma

#### Anticipated stigma

Whether FGD participants disclosed their diagnoses differed per disease type. All participants with CL or CD had told their families; in contrast, several participants affected by leprosy did not tell anyone about their disease out of fear of rejection: ‘*That is why I didn't tell my husband, because everything at my home is great, and I love him and he loves me as it was when we got to know each other. I trust him, but regarding this, no!’* [G1 – F, 55, leprosy]

The interviews confirmed these findings; all but two participants said they preferred that others did not find out they had leprosy. Five people explicitly mentioned not wanting other people to know because they feared rejection. One explained how she was afraid it would affect her family income:

I would not like if they saw my face on television because my husband sells pastries, so the people would say, ‘No, now I'm not going to buy any more!’ Because people are like that; people don't understand others [I2 – F, 44, leprosy].

Four interviewees said to invent excuses if people ask about symptoms, such as an allergy, a bacterial infection or even cancer. This topic also came up in the FGD: one participant said she tells others she has dermatitis, and another person told colleagues that the darker color of his skin, a result of the medication, was caused by the sun.

Most people with CL and CD did not mention any rejection from their communities and therefore did not attempt to conceal their diseases. The participants from FGDs 3 and 4 described the disease as ‘common’ and ‘normal’. However, one man said that although CL was very common in the countryside, it does cause stress and tension in the area surrounding the farm where he works due to prejudice and the fear of spreading infection. Therefore, others avoid infected persons for a certain period of time.

#### Experienced stigma

Several interviewees affected by leprosy (*n* = 8) never felt rejected. Two of them explicitly mentioned that this was because almost nobody knew they were suffering from the disease, while others felt support from family, neighbors and friends.

Five respondents affected by leprosy had experienced rejection or discrimination by others. Two persons spoke about experiencing discrimination by family members. This also came up in the FGDs. One woman told that her sister initially rejected her but came around when she found out leprosy is curable. A woman in FGD 1 said that her husband left her after her diagnosis. Similarly, an interviewee reported that his brother-in-law told him that he could not leave the house having leprosy.

For those with leprosy, the experience of discrimination was a major factor affecting work life. Although participants from all disease groups initially concealed their illness from their coworkers and supervisors, some were detected by colleagues: ‘*They were asking me, “Why are you so dark?”, and a friend had seen a health poster, which said it came from a white lesion on the skin, and he said to me, “Is it that what you have?”, and from then* [*on*] *they started to discriminate* [*against*] *me’* [G1 – M, 40, leprosy].

A common experience of rejection by medical personnel revealed that not all healthcare workers have sufficient knowledge about leprosy, which has led to the refusal to treat patients, not wanting to touch patients and putting patients in isolation rooms in hospitals: ‘*He kept looking at the clinical history, and then he went: “Aye, a patient with leprosy” … everybody got scared, and then immediately they put on a mask, they put on gloves*’ [G2 – M, 64, leprosy].

These findings were confirmed in the interviews. When a woman affected by leprosy went for a biopsy, the secretary loudly said, ‘Ah, the biopsy for patients with leprosy’, and all the other people looked at the woman and moved away. Another person felt rejected by a bacteriologist, who refused to perform a test, and by a doctor who only had to do a check-up but was dressed as if he had to perform surgery.

An FGD participant with CD shared a negative experience with a healthcare worker when he wanted to donate blood:

‘The nurse told me: “Your blood doesn't serve. We are going to burn it, and you are going to die of Chagas; look and go to your health insurance”’ [G1 – M, 55, CD].

#### Internalized stigma

An interviewee mentioned that she did not tell anyone about having leprosy, but she felt she needed to isolate herself from her loved ones, and left her husband without telling him about her leprosy. A man mentioned that he had never been discriminated against by others but that his self-confidence had declined because he always used to be healthy and active, and leprosy did not allow that anymore. Likewise, another man shared how he was previously very active and felt that the disease had rendered him useless.

## Discussion

This explorative qualitative study aims to improve understanding of the context of the PSB of CD, CL and leprosy reported in the related study by Gómez *et al*. (2020). The high levels of mental distress reported by persons affected by CD could be explained by impairments and stress related to the disease. For persons affected by CL in our study sample, the treatment had a serious impact on their mental well-being. In persons affected by leprosy, the burden was related primarily to anticipated and experienced stigma.

For people affected by CD, mental distress consisted of the fear of dying and feelings of frustration and uselessness because they could not perform their work. This is in line with a relatively high score on the SRQ-20 scale for mental distress found in the study by Gómez *et al*. (2020). Ozaki *et al*. (2011) and da Silva *et al*. (2010) also showed that depressive symptoms are often found in persons affected by CD: ‘Chagas disease provokes a social uncertainty that has a strong mental impact on patients, particularly because there is no cure for this disease’ (da Silva et al., 2010, p. 10). Participation restrictions appear to be mainly caused by disease- and treatment-related impairments and by physical activity limitations, such as walking longer distances. Lima-Costa *et al*. (2005) found a relationship between satisfaction with social network and self-rated health in elderly persons with CD in Brazil. Additionally, worse self-rated health was an indicator of psychological distress, in line with the findings of this study. Contrary to the current study, Ozaki *et al*. (2011) found CD-related stigma related to work, especially when Brazilian workers were applying for jobs. This led to discrimination by employers based on a fear of reduced labor output due to CD-associated impairments.

People affected by CL reported frustration and stress related to treatment and restrictions in participation in family life and work. These feelings and restrictions decreased after recovery. People affected by CL did not share feelings of rejection or other indications of stigmatization. CL has frequently been associated with depressive symptoms and severe participation restrictions (Yanik *et al*., 2004; Reithinger *et al*., 2005; Kassi *et al*., 2008). However, this seems to depend on the cultural setting and the location of the skin lesions, as also emphasized by Gómez *et al*. (2020). Levels of stigma and discrimination typically correlate with the visibility of lesions and scars (Hofstraat and van Brakel, 2016; Al-Kamel, 2017). Although these clinical features were not systematically captured in the current study, few participants reported lesions in their face or on their hands.

The current study found that most people affected by leprosy choose to conceal their condition from others due to fear of rejection. This is in line with findings from other countries, such as India, Indonesia and Nepal (Adhikari *et al*., 2014; Das *et al*., 2015; Indow *et al*., 2019; Rai *et al*., 2020). Most participants in the current study did not report severe examples of experienced stigma or internalized stigma, whereas, in Nigeria and Indonesia, it was found that most people suffering from leprosy feel very ashamed (Ebenso *et al*., 2019; Indow *et al*., 2019). Furthermore, in Indonesia and Ghana, most leprosy patients felt stigmatized by healthcare providers, in the labor market and by the community (Oduro, 2017; Rai *et al*., 2020), whereas, in this study, only a minority reported these experiences. Possibly, the disease impairments were less visible due to earlier diagnosis and better access to health care in the researched areas (mostly urban) and therefore more concealable. This indicates that the potential for stigma would still exist (Goffman, 2009).

The results reported in this study are related to physical impairments as well as stigma. They therefore address the need for physical rehabilitation, including self-care, as well as social and psychological aspects to improve the well-being of persons affected by these NTDs. Strengthening social support can contribute to reducing stigma and improving mental well-being and social participation (Baumeister and Leary, 1995; Connell *et al*., 2012). Additionally, policies that strengthen the position of an employee and prevent job loss based on health discrimination would provide valuable benefits (Sanmartino *et al*., 2015). Furthermore, this study emphasizes the need for raising awareness in society to prevent stigmatization, worse mental well-being and restrictions in participation. Further investigation and analysis on a larger scale are urgently needed to better understand the full dimensions of the burden of NTDs and what interventions are suitable and effective.

This research is one of the first studies to research social factors associated with CD. One limitation is that data analysis was performed by non-native Spanish speakers. This may have caused interpretation bias, and important information could have been overlooked. However, findings were discussed with the Colombian researchers when doubt existed, and general agreement was reached regarding the reported results.

A further limitation is the lack of data on the severity of the participants' disease manifestations, as this information is not routinely captured in the respective national program registries that served as the researchers' clinical information source. Data on disease severity would allow more detailed insight into the reported burden, leading to more valid conclusions when comparing the results across the three NTDs.

## Conclusions

We described the type and qualitative dimensions of mental distress, participation restriction and stigma found in people affected by leprosy, CL and CD in two co-endemic areas of Colombia, complementing the quantitative study by Gómez *et al*. (2020). Persons affected by all three diseases reported that their health condition negatively affects their mental well-being and social participation. Persons affected by leprosy also reported severe forms of stigma that impact their lives. The findings indicate that these diseases impose a significant PSB on patients in the studied regions. Efforts to eliminate NTDs should not only focus on the elimination of infection, but also address the impact on mental well-being and participation caused by impairments, disability and stigma. Consistent data collection on the PSB and the sharing of knowledge about effective interventions will contribute to the holistic approach needed to win the fight against NTDs.

## Appendix

### Appendix A: Focus group discussions guide

**General aspects of the focus group discussions:** They took place on 15 and 16 May at the university Francisco de Paula Santander in Cúcuta and on 15 June at the institute of Mission Médica in Arauca. In total, four groups were organized on the three pathologies.

**Themes:** Family, work and community.

**Activities**

• Greetings and acquaintance.
• Sharing of experiences in the light of the three themes.
• Selection and prioritization of three experiences which will be further discussed.
• Group discussion on each of the preselected topics:
  ◦ What do you think of when you hear this story/experience?
  ◦ Why (or why not) do you identify yourself with this story?
  ◦ What makes this experience problematic?
  ◦ How can this situation be prevented?
• Closing of the meeting, reflection on the process and participation.

### Appendix B: Interview topic list

• Moment of diagnosis: experiences and emotions
• Disease disclosure and support
• Impact on life: participation in family, work and community
• Experience of rejection by others
• Emotional impact on self-image
