# Psychosocial burden of neglected tropical diseases in eastern Colombia: an explorative qualitative study in persons affected by leprosy, cutaneous leishmaniasis and Chagas disease – ERRATUM

**DOI:** 10.1017/gmh.2021.31

**Published:** 2021-08-05

**Authors:** Robin van Wijk, Lena van Selm, Martha C. Barbosa, Wim H. van Brakel, Mitzi Waltz, Karl Philipp Puchner

The publisher apologises that upon publication the authors names were incorrectly listed within the citation information

The correct author information is:

van Wijk R, van Selm L, Barbosa MC, van Brakel WH, Waltz M, Puchner KP (2021).

## References

[ref1] Van Wijk R, Van Selm L, Barbosa M, Van Brakel W, Waltz M, and Puchner KP (2021) Psychosocial burden of neglected tropical diseases in eastern Colombia: An explorative qualitative study in persons affected by leprosy, cutaneous leishmaniasis and Chagas disease. Global Mental Health, 8, E21. doi:10.1017/gmh.2021.18 34249368 PMC8246647

